# Treating iron overload in patients with non-transfusion-dependent thalassemia

**DOI:** 10.1002/ajh.23405

**Published:** 2013-02-06

**Authors:** Ali T Taher, Vip Viprakasit, Khaled M Musallam, M Domenica Cappellini

**Affiliations:** 1 Department of Internal Medicine, American University of Beirut Medical CenterBeirut, Lebanon; 2 Department of Pediatrics and Siriraj-Thalassemia Center, Faculty of Medicine, Siriraj Hospital, Mahidol UniversityBangkok, Thailand; 3 Department of Medicine and Medical Specialities, Universitá di MilanoCa' Granda Foundation IRCCS, Milan, Italy

## Abstract

Despite receiving no or only occasional blood transfusions, patients with non-transfusion-dependent thalassemia (NTDT) have increased intestinal iron absorption and can accumulate iron to levels comparable with transfusion-dependent patients. This iron accumulation occurs more slowly in NTDT patients compared to transfusion-dependent thalassemia patients, and complications do not arise until later in life. It remains crucial for these patients' health to monitor and appropriately treat their iron burden. Based on recent data, including a randomized clinical trial on iron chelation in NTDT, a simple iron chelation treatment algorithm is presented to assist physicians with monitoring iron burden and initiating chelation therapy in this group of patients. Am. J. Hematol. 88:409–415, 2013. © 2013 Wiley Periodicals, Inc.

## Introduction

The thalassemias are a group of inherited disorders that are caused by altered or absent hemoglobin chain synthesis leading to ineffective erythropoiesis and subsequent anemia. The symptoms of these diseases can vary substantially in severity 1–6. Some patients, like those with the carrier genotypes, have no clinically obvious symptoms. Others, like β-thalassemia major patients, depend on lifelong transfusions for survival.

Non-transfusion-dependent thalassemia (NTDT) is a recently introduced term used to describe those thalassemia phenotypes that do not require regular blood transfusions for survival. Patients with very severe HbE/β-thalassemia may require regular blood transfusions 7, but these and other patients requiring regular transfusions would not be considered NTDT patients for the purposes of treating iron overload. Patients who are dependent on transfusions, regardless of genotype, would be managed as β-thalassemia major patients.

NTDT comprises a range of hemoglobin disorders including β-thalassemia intermedia, α-thalassemia (mainly HbH disease), HbE/β-thalassemia, HbS/β-thalassemia, and HbC thalassemia. The most prevalent forms of NTDT worldwide are HbH disease, HbE/β-thalassemia, and β-thalassemia intermedia 8. NTDTs primarily exist in low- or middle-income countries of the tropical belt stretching from sub-Saharan Africa, through the Mediterranean region and the Middle East, to South and Southeast Asia 8. However, recent global population movements have also led to increasing incidence in areas of the world previously relatively unaffected by these conditions, such as North Europe and the Americas 9, 10.

Clinical features of NTDT are variable, making diagnosis of NTDT challenging. Both the β-thalassemia intermedia and HbE/β-thalassemia phenotypes show a wide spectrum of disease severity 11, 12. Patients may have a very mild phenotype and normal growth, or may exhibit severe anemia, growth retardation, hypersplenism, and a variety of morbidities that may eventually require regular transfusion therapy 13. Patients with HbH disease present with anemia, splenomegaly, jaundice, and growth retardation 14–16. Patients who have HbH along with the Constant Spring or Paksé mutation have a much more severe clinical phenotype 15, 17. Several genetic and environmental factors are known to modify phenotype in NTDT; however, the diagnosis remains largely clinical and is based on the severity of the patient's condition.

Early diagnosis and monitoring of NTDT are critical to ensure appropriate and timely treatment of symptoms and to prevent serious complications later in life. Complications associated with NTDT may be as serious as those observed in β-thalassemia major. However, since patients with NTDT usually have a milder and more slowly progressing phenotype than β-thalassemia major patients have, there is a risk that regular monitoring and treatment may be delayed until complications become obvious.

Many complications associated with thalassemia are related to excessive iron accumulation. Although patients with NTDT do not depend on regular transfusions, their intestinal iron absorption is increased 18, 19. Iron accumulation in NTDT patients occurs more slowly than in transfusion-dependent patients 19, but can pose a serious risk to the patients' health. Iron overload in untransfused β-thalassemia intermedia patients has been estimated at 1.0–3.5 g/year 19, compared with 2.0–12.0 g/year in regularly transfused patients 20, 21. NTDT patients may have extensive liver iron loading that is disguised by a relatively low serum ferritin level compared to what would be seen in transfusion-dependent patients 22–24. In addition, current thresholds used to guide chelation therapy in transfusion-dependent patients are based on the association between serum ferritin/liver iron concentration (LIC) and cardiac complications or death 25. However, siderotic cardiac disease and secondary death do not seem to be a concern in NTDT patients 26–30. The current standard thresholds of serum ferritin and LIC for estimating risk of complications in β-thalassemia major can therefore not be extrapolated to NTDT patients. This is a key challenge for assessing and treating iron overload in NTDT patients.

NTDT patients may require iron chelation to address the risk of iron overload. Unlike in β-thalassemia major, there are few data for iron overload and chelation therapy in β-thalassemia intermedia, and even fewer for HbH disease and HbE/β-thalassemia. Recent data, including a randomized investigational trial of iron chelation in the NTDT population 18, have prompted a revision of a previous iron chelation treatment algorithm 31 to incorporate novel findings and their interpretation. The purpose of this review is to provide an overview of the challenges and complications associated with NTDT and to present a practical decision-making algorithm for monitoring and treating iron overload in NTDT patients. Up-to-date clinical recommendations will support approaches to monitoring iron burden and initiating chelation therapy in NTDT patients.

### Iron overload in non-transfusion-dependent thalassemia patients

NTDT patients are susceptible to iron overload, although the mechanism of iron accumulation is quite different from that observed in β-thalassemia major patients 32. Whilst NTDT patients receive no or only occasional transfusions, their intestinal iron absorption is continuously upregulated, leading to slow accumulation of iron in tissues, particularly in the liver 23, 33. The mechanism of increased intestinal iron absorption in NTDT patients is triggered by a cascade initiated by ineffective erythropoiesis, which is characteristic of these diseases 33, 34.

The anemia and hypoxia resulting from ineffective erythropoiesis influence the expression of the serum protein hepcidin, which is a key regulator of intestinal iron absorption 35, 36. Hepcidin negatively regulates iron absorption because it downregulates the expression ferroportin, a transmembrane protein responsible for exporting intracellular iron into circulation and for iron absorption from the gastrointestinal tract (GIT) 37. Hepcidin levels decline when iron sequestration for erythropoiesis increases 35, and this, in turn, results in upregulated ferroportin. High levels of ferroportin cause an increased release of iron from macrophages and increased iron absorption from the GIT 23, 38. In NTDT, downregulation of hepcidin is mediated by extensive erythropoiesis as well as chronic anemia 38, hypoxia 38, as well as growth differentiation factor-15 (GDF-15) 39, 40 and twisted gastrulation factor 41. However, recent data show that GDF-15 is not essential for systemic iron homeostasis in mice 42, Also, the role of hypoxia in iron overload is not well understood considering that other disease entities where hypoxia is a prominent feature do not show evidence of substantial iron overload (e.g., pyruvate kinase deficiency). It is clear that much research is still needed to elucidate the exact mechanism underlying iron overload in NTDT.

### Why iron overload matters: complications

Complications in NTDT patients result from a number of factors, primarily ineffective erythropoiesis, chronic anemia, and hemolysis, and iron overload 11, 14, 43. Although some commonalities exist, the range of complications seen in patients with NTDT is distinct from those observed in the transfusion-dependent thalassemias 44.

Complications common to different types of NTDT include extramedullary hematopoiesis, thrombosis, and pulmonary hypertension (PHT), leg ulcers, hepatic disease and hepatocellular carcinoma (HCC), cholelithiasis, endocrinopathies, and bone disease 15, 45–47. The rate of many of these complications increases with age 48. Nevertheless, it is thought that increased iron accumulation underlies some of these complications or contributes in some way to their severity 48. Observational studies have reported positive associations between iron overload and various morbidities in NTDT. Musallam *et al* recently found a strong correlation between the rate of change in serum ferritin level and the rate of change in transient elastography values (a measure of hepatic stiffness predictive of fibrosis) in a group of non-transfusion-dependent patients with β-thalassemia intermedia 49. The results from this study clearly show that in NTDT, decreases in serum ferritin by means of iron chelation are associated with improvements in measures of hepatic fibrosis. There is also evidence of HCC in patients with NTDT 50, 51. Hepatic manifestations of iron overload in NTDT therefore appear to resemble the reports of hepatic complication due to iron overload in patients with hereditary hemochromatosis and β-thalassemia major 52–54.

An association between iron overload and endocrine/bone disease was also observed in a cross-sectional study that recruited 168 patients with β-thalassemia intermedia, especially those with a LIC ≥6 mg Fe/g dw 55, further echoing data from β-thalassemia major patients 56. Ineffective erythropoiesis and age could still be potential confounders for the association between iron overload and osteoporosis. However, after adjustment for both risk factors in the aforementioned study, the association between iron overload and osteoporosis persisted. Evidence for a toxic role of iron on bone metabolism does exist 57. Also, a study from Thailand showed by means of bone histomorphometric analyses that suboptimally transfused thalassemia patients with osteopenia and osteoperosis have impaired bone matrix maturation, defective mineralization and focal iron deposition. In this study 12 of the 17 enrolled patients had NTDT (HbE/β-thalassemia) 58.

There is also evidence for an association between iron overload and vascular disease in NTDT patients. Increased LIC was associated with a higher prevalence of thrombosis and PHT in a cross-sectional analysis of β-thalassemia intermedia patients 55, and in splenectomized adults there is a relationship between iron overload and cerebrovascular disease 59, 60. Although these associations persist after adjustment for potential confounders such as age and severity of disease, we believe it is more likely that in NTDT patients hypercoagulability and endothelial damage are the main contributors in the development of vascular complications.

Further molecular-, radiologic-, and longitudinal-studies, designed to assess to causal relationships between iron overload and certain morbidities in NTDT patients, is needed. Nevertheless, elevated LIC in NTDT, per se, is a pathologic feature that warrants treatment.

### Measuring iron overload in NTDT patients

As iron overload is a risk to the health of NTDT patients, reliable, and accurate methods of monitoring body iron levels are essential. It is especially important in NTDT patients; because they do not receive regular blood transfusions, transfusion history cannot be used to estimate iron burden as it is in β-thalassemia major patients. Therefore, direct or indirect measurements of body iron should be used. Measurements of iron status are used to make treatment decisions and to measure patients' progress with therapy. The choice of method for measuring iron accumulation depends on both the patient's needs and on the available facilities.

### Serum ferritin

**Figure 1:**
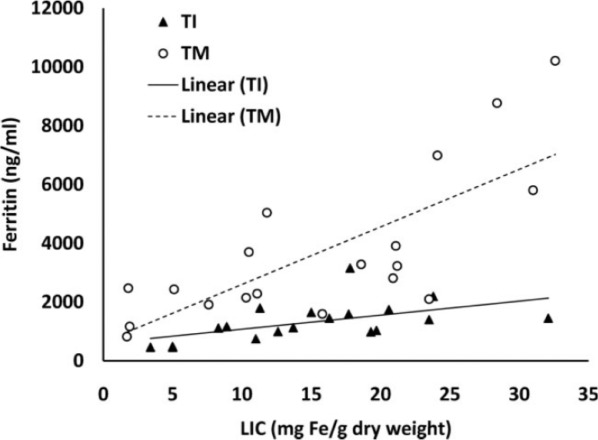
Linear regression analysis of serum ferritin versus liver iron concentration (LIC) in both β-thalassemia intermedia and β-thalassemia major patients. The 95% confidence intervals for slope β-thalassemia intermedia are [17.0–68.2] and for slope β-thalassemia major are [109–283]. As the error bars do not overlap, the slope differences are statistically significant. There are no differences in the intercepts. (Reproduced with permission from Taher et al. Am J Hematol 2010; 85:288–290.)

Estimations of body and liver iron can be made by measuring serum ferritin by a simple blood test 61, 62. This method is inexpensive and accessible. However, caution must be exercised when interpreting serum ferritin values, especially in NTDT patients. While the correlation between serum ferritin and liver iron has been established in β-thalassemia major 63, 64, the relationship has been shown to be quite different in NTDT 22. In NTDT patients, where iron accumulation occurs through increased dietary absorption rather than from blood transfusions 23, 24, liver iron may be much higher for a given serum ferritin value, compared to what would be expected for a β-thalassemia major patient. Clinical studies have compared LIC and serum ferritin levels in β-thalassemia intermedia 22–24, 30 and HbE/β-thalassemia 24 patients with those in β-thalassemia major patients. All studies found that, at a comparable LIC level, serum ferritin levels in the NTDT patients were significantly lower 22–24, 30. Although the association between serum ferritin levels and LIC is significantly different in NTDT patients, a relationship does exist. A significant positive correlation between serum ferritin and LIC has been seen in all of the main types of NTDT 15, 18, 22, 24. Figure 1 shows the linear regression analysis of serum ferritin versus LIC in both β-thalassemia intermedia and major patients enrolled in a study by Taher *et al* 30. Despite having comparable LICs, transfusion-independent patients in this study had significantly lower serum ferritin than β-thalassemia major patients. Serum ferritin had a statistically significant steeper (nearly fivefold) relationship with LIC in β-thalassemia major compared with β-thalassemia intermedia. While LIC is preferred as a measurement of iron overload, serum ferritin can still be used in the clinical setting to estimate LIC and overall iron burden if necessary.

**Figure 2:**
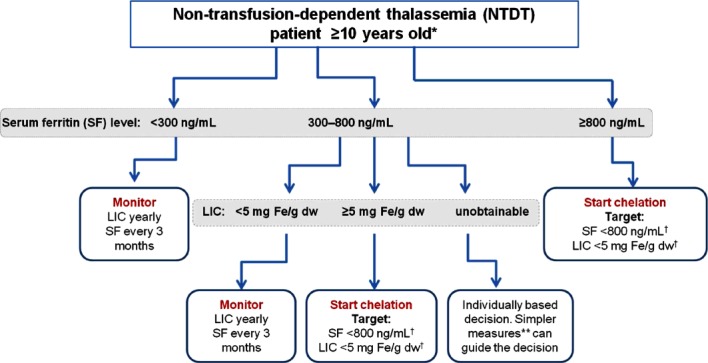
Proposed treatment algorithm for iron overload in NTDT. This simple algorithm presents serum ferritin and LIC thresholds for initiating and stopping chelation therapy in NTDT patients based on current evidence. *Because patients with HbH disease typically accumulate iron much more slowly, serum ferritin and LIC monitoring can begin at 15 years instead of 10 years. ^†^Iron chelation should be stopped at LIC of 3 mg Fe/g dw, or SF of 300 ng/mL, as safety data are not available to support continued chelation below this level. ** I.e. ascorbic acid and serum transferrin levels, changes in marrow space observed during clinical examination or by means of X-ray, assessment of liver spleen size plus height, hemoglobin concentrations. [Color figure can be viewed in the online issue, which is available at http://wileyonlinelibrary.com.]

### Liver iron biopsy

The liver is the primary site of iron accumulation for NTDT patients, highlighting the importance of accurate assessment of LIC. LIC can be measured directly by needle biopsy; however, due to risks with the procedure this is not the preferred method. The most common adverse event with liver biopsy is pain at the needle site. More serious complications can include hemorrhage or sepsis, although these are rare 65. Liver iron accumulation has been shown to be uneven in β-thalassemia 66 and cirrhosis 67, 68, resulting in a risk of sampling error 66–68. Furthermore, different tissue processing methods can produce variable LIC measurements 69.

### Magnetic resonance imaging

Magnetic resonance imaging (MRI) using either R2 (1/T2) or R2* (1/T2*) pulse sequences is a reliable and noninvasive method for assessing LIC, and has been validated against liver biopsy measurements 70–76. Iron overload can be measured in NTDT patients using the same techniques as for patients with other types of iron overload, including hereditary hemochromatosis or transfusion-related iron overload. In one study validating R2* MRI measurement in patients with iron overload, R2* MRI values were strongly correlated with LIC values from liver biopsy (*r*_s_ = 0.96–0.98, *P* < 0.001) 70. In another study of R2 and R2* MRI measurements in regularly transfused iron-overloaded patients including those with β-thalassemia major and intermedia, LIC as measured by biopsy maintained a linear correlation (r=0.97) with R2* MRI up to 32.9 mg Fe/g dry weight (dw) 71. Previous studies have reported strong correlations for R2 72, T2 73, 74 and T2* 75 measurements with liver iron by biopsy, confirming that MRI is a reliable and accurate way of measuring LIC in iron-overloaded patients. The upper limit to reliably estimate LIC by MRI is approximately 30–40 mg/g dw, depending on the scanner specifications 77. T2* MRI may be used to accurately measure iron concentration in the heart 78; however, cardiac iron overload is not typically seen in NTDT patients 26, 28–30. The benefits of measuring LIC by MRI are clear; unfortunately, MRI machines are not always readily available in facilities where NTDT patients are treated 76.

### Other liver iron quantification methods

Devices that estimate the magnetic susceptibility can also be used to quantify LIC noninvasively. The Superconducting Quantum Imaging Device (SQUID) and the Magnetic Iron Detector (MID) are such devices. However, devices with superconducting magnets like SQUID are expensive and this kind of equipment is therefore only available in a few centers worldwide 79. In addition, SQUID is not particularly accurate for measurements of LIC ranging between 3 and 10 mg/g dw. Newer devices, such as the room-temperature MID offer promise for low-cost, non-invasive quantification of LIC in the future.

### Treating iron overload: Iron chelation in NTDT

Iron overload can be managed with iron chelation therapy. Clinical studies, in particular the recent THALASSA 18 trial, have shown that iron chelation is effective for reducing liver iron and serum ferritin in NTDT patients 51, 80–85. A summary of studies of iron chelation therapy in patients with NTDT is presented in Table 1. Previous investigational studies have shown reduction in serum ferritin in transfusion-independent HbE/β-thalassemia patients with deferiprone treatment 81, 84, and in β-thalassemia intermedia patients with subcutaneous deferoxamine therapy 85. These studies have laid the groundwork for the treatment of iron overload in NTDT, showing that NTDT patients do have a chelatable iron pool 80, 85 and that measures of iron overload may be improved with chelation 51, 80–85. Iron chelation with deferasirox and deferiprone has also been shown to be generally well tolerated in NTDT patients 82, 86, 87.

**TABLE 1:** Overview of the Investigational Use of Iron Chelation Therapy in NTDT (by Therapy in Reverse Chronological Order)

		Description				
			
Study	Disease type	Drug investigated	*N* (patient ages, years)	Type of study	Study objectives	Results
*Deferasirox*						
Thalassa 18 Taher et al. Blood 2012;120:970–977	NTDT	Deferasirox (starting dose 5 or 10 mg/kg/day)	166 (≥10)	Prospective, randomized, double-blind, placebo-controlled trial	Efficacy (measured by change in LIC and SF) and safety of deferasirox over 52 weeks in iron overloaded patients with NTDT	Significant^a^ decrease in both LIC and SF after 12 months, compared to placebo
Ladis et al. Br J Haematol 2010; 151:504–508 83	β-TI	Deferasirox (starting dose 10 or 20 mg/kg/day)	11 (25–40)	Prospective, single-arm, open-label trial	Efficacy (measured by changes in hepatic and cardiac iron, and SF) and safety of deferasirox to 24 months	Significant^a^ decrease from baseline in LIC and SF after 12 and 24 months
Voskaridou et al. Br J Haematol 2010; 148:332–334 88	β-TI	Deferasirox (starting dose 10 or 20 mg/kg/day)	11 (28–53)	Prospective, single-arm, open-label trial	Efficacy and safety of deferasirox in sporadically transfused, iron overloaded patients with β-TI over 12 months	Significant^a^ improvement in both liver T2* and mean SF after 12 months
*Deferiprone*						
Akrawinthawong et al. Hematology 2011; 16:113–122 81	HbE/ β-thal	Deferiprone (starting dose 50 mg/kg/day)	30 (18–50)	Prospective, single-arm, open-label trial	Efficacy of deferiprone in reducing possibility of cardiac complications over 1 year in HbE/ β-thal patients receiving intermittent transfusions	Significant^a^ decrease in mean pulmonary arterial pressure and pulmonary vascular resistance and significant^a^ decrease in SF after 1 year
Chan et al. Br J Haematol 2006; 133:198–205 82	HbH disease	Deferiprone (starting dose 50 mg/kg/day)	17 (29–76)	Prospective, control-matched, open-label trial	Efficacy and toxicity of deferiprone in HbH patients with gross iron overload over 18 months, compared with age- and HbH genotype-matched controls without iron overload	Significant^a^ reduction in SF after 6 and 18 months
Pootrakul et al. Br J Haematol 2003; 122:305–310 84	HbE/ β-thal or β-TI	Deferiprone (starting dose 25 or 50 mg/kg/day)	9 (20–48)	Prospective, single-arm, open-label trial	Efficacy and toxicity of deferiprone over 17–86 weeks	Significant^a^ decreases in SF, LIC, red cell membrane iron and NTBI; reduced transfusion requirements in four patients
Rombos et al. Haematologica 2000; 85:115–117 89	β-TI	Deferiprone (75 mg/kg/day)	3 (>18)	Prospective, single-arm, open-label trial	Efficacy (change in SF and urinary iron excretion) and safety of deferiprone over 2 years	Decline in SF in all patients within 6 months and was maintained over 24 months; arthropathy and agranulocytosis were not observed
Olivieri et al. Blood 1992;79:2741–2748 90	β-TI	Deferiprone (75 mg/kg/day)	1 (29)	Case study	Change in iron status of a 29-year-old man with deferiprone treatment over 9 months	Decrease in SF from 2174 ng/mL to 251 ng/mL after 6 months; Decrease in LIC from 14.6 mg Fe/g dw to 1.9 mg Fe/g dw after 9 months
*Deferoxamine*						
Pippard and Weatherall. Birth Defects 1988;23:29–33 85	β-TI	Deferoxamine (150 mg/kg over 24 hours)	4 (18–27)	Prospective, placebo-controlled crossover trial	Effect of deferoxamine on iron balance in β-TI patients with positive iron balance	All patients achieved negative iron balance after a 6 days of deferoxamine treatment
Cossu et al. Eur J Pediatr 1981; 137:267–271 80	β-TI	Deferoxamine (3 days each of 20, 40, 60, 80 and 100 mg/kg/day)	10 (1.2–17.3)	Prospective, single-arm open-label trial	Urinary iron excretion over 24 hours and change in SF over 6 months	Significant^a^ increases in urinary iron excretion; non-significant decreases in SF

a *P* ≤ 0.05; SF, serum ferritin; β-TI, β-thalassemia intermedia.

Until the THALASSA trial, most studies investigating the safety and efficacy of iron chelation therapy in NTDT were small, open label and single arm, limiting their applicability in wider populations. In contrast, THALASSA was a randomized, double-blind, placebo-controlled trial that evaluated the safety and efficacy of iron chelation for investigational use over 1 year in a large cohort of NTDT patients. 18. The study included patients with β-thalassemia intermedia, HbH disease and HbE/β-thalassemia. Approximately 90% of the patients had previously received blood transfusions, but none had received a transfusion within 6 months of beginning the study. Patients received either placebo or the iron chelator deferasirox at starting doses of 5 mg/kg/day or 10 mg/kg/day, with dose escalations up to 20 mg/kg/day. The study found a significant decrease in LIC after 1 year of treatment. This decrease was proportional to the dose of chelation they received, and both dosage groups experienced decreases significantly greater than the placebo groups. Similar results were observed for serum ferritin levels 18. The study also confirmed that deferasirox had a manageable safety profile, with a similar overall adverse event incidence for the deferasirox groups and placebo. The main drug-related adverse events were gastrointestinal; however, frequency of these was similar between the treatment and placebo groups 18.

### Treating iron overload: Proposed treatment algorithm

The decision to initiate iron chelation in NTDT patients depends on the estimated extent of iron overload in each patient (Figure 2 shows a proposed treatment algorithm for iron overlaod in NTDT). Body iron monitoring should begin when the patient is 10 years old 18. The following applies to alpha-thalassemia, especially HbH disease which can be devided into deletional (–/–α) and non-deletional (–/αTα or –/ααT) type: Patients with non-deletional mutations usually have a more anemic phenotype than deletional HbH and they may require infrequent blood transfusions and/or splenectomy. A decision with regards to iron monitoring in non-deletional HbH should therefore depend on a patients' blood transfusion history. In general, patients with deletional type HbH typically accumulate iron much slower than other NTDT patients 6, 15, 16 and monitoring can begin at 15 years of age.

Measuring serum ferritin is a simple method that may act as a surrogate to estimate the extent of total iron load. We recommend that chelation should be initiated if serum ferritin rises above 800 ng/mL, with the objective of reducing levels to 300 ng/mL 91. Chelation therapy dosing should be titrated if serum ferritin continues to rise, or if it falls too quickly. Serum ferritin should also be used for monitoring patients' progress over the course of chelation therapy.

When serum ferritin levels are between 300 ng/mL and 800 ng/mL 91, LIC should be measured to more accurately determine the extent of iron overload. LIC can be measured directly, either by biopsy or by a non-invasive method such as MRI. Initiation of chelation therapy should be started at a LIC of 5 mg Fe/g dw 55. Previous treatment algorithms have suggested a LIC threshold of 7 mg Fe/g dw 31, 92 for initiating chelation. However, recent evidence showing increased prevalence of morbidities at LIC of 6-7 55 and ≥5 mg Fe/g dw [Bibr b93], has prompted this revised recommendation. Chelation therapy should be initiated at LIC ≥5 mg Fe/g dw in order to prevent complications before they develop. If direct measurement of LIC is not available, then the decision to initiate chelation should be made on an individual basis and should be guided by the treating physicians' opinion.

Data from the THALASSA trial show that a cut-off serum ferritin level of <300 ng/mL was highly predictive of LIC <3 mg Fe/g dw 91. An LIC of 1–2 mg Fe/g dw is considered normal 94, 95, and there are no data to support the safety of iron chelation below 3 mg Fe/g dw. Therefore, iron chelation therapy should be stopped when serum ferritin reaches 300 ng/mL or when LIC reaches 3 Fe/g dw. When body iron levels fall below these thresholds, the patient's iron levels should be monitored regularly. Serum ferritin should be measured monthly and LIC should be measured yearly to identify iron accumulation at potentially toxic levels.

Reductions in serum ferritin and LIC with iron chelation can be expected to be accompanied by similar reductions in morbidity and complications in NTDT patients due to the reduced iron load 55, 81, 93. Further studies of the benefits of chelation on long-term complications of iron overload and survival are warranted.

## Conclusions

Although patients with NTDT are generally considered to have a less severe form of thalassemia compared with patients with transfusion-dependent disease, over time, they can achieve similar levels of liver iron. By definition, NTDT patients are not dependent on blood transfusions for their survival. They may receive occasional transfusion therapy; for example, in the case of growth retardation, infection, severe anemia, or pregnancy 96. In general, the main contributor to total iron burden is increased intestinal absorption rather than blood transfusions.

Adequate assessment, monitoring and iron chelation treatment of NTDT patients are crucial for preventing the complications known to be associated with increased iron burden. Recent advances in the understanding of the mechanisms of iron overload in NTDT patients and the relationship between LIC and serum ferritin have prompted a need for re-evaluation of previous treatment recommendations. There are currently no standard clinical practice guidelines for the treatment of iron overload in NTDT patients.

Recent randomized trial data showing the efficacy and manageable safety profile of iron chelation in NTDT support the use of iron chelation therapy the NTDT patient population. Revision of previous treatment guidelines is necessary to reflect these recent advances and provide further guidance for physicians who care for NTDT patients.
